# The Appropriate Opportunity for Evaluating Liver Fibrosis by Using the FIB-4 Index in Patients with Nonalcoholic Fatty Liver Disease in Japan

**DOI:** 10.3390/diagnostics10100842

**Published:** 2020-10-19

**Authors:** Yuya Seko, Kota Yano, Aya Takahashi, Shinya Okishio, Seita Kataoka, Keiichiroh Okuda, Atsushi Umemura, Kanji Yamaguchi, Michihisa Moriguchi, Saiyu Tanaka, Yoshito Itoh

**Affiliations:** 1 Department of Molecular Gastroenterology and Hepatology, Kyoto Prefectural University of Medicine, Kyoto 6028566, Japan; yanokota@koto.kpu-m.ac.jp (K.Y.); ayataka@koto.kpu-m.ac.jp (A.T.); okishin@koto.kpu-m.ac.jp (S.O.); s1120@koto.kpu-m.ac.jp (S.K.); k-okuda@koto.kpu-m.ac.jp (K.O.); aumemura@koto.kpu-m.ac.jp (A.U.); ykanji@koto.kpu-m.ac.jp (K.Y.); mmori@koto.kpu-m.ac.jp (M.M.); yitoh@koto.kpu-m.ac.jp (Y.I.); 2 Center for Digestive and Liver Diseases, Nara City Hospital, Nara 6308305, Japan; saiyusun@yahoo.co.jp

**Keywords:** FIB-4 index, nonalcoholic fatty liver disease, liver fibrosis

## Abstract

In patients with nonalcoholic fatty liver disease (NAFLD), liver fibrosis is the predictive factor for liver-related events and prognosis. This retrospective study aimed to evaluate longitudinal changes in the FIB-4 index and to determine a strategy for diagnosing and following patients with NAFLD using this index. We analyzed the FIB-4 index at baseline and after 1 and 5 years in 272 consecutive patients with biopsy-proven NAFLD. Of these, 52 patients underwent serial biopsies. The change in the FIB-4 index was correlated with changes in the fibrosis stage among these patients (*p* = 0.048). The median FIB-4 index was 1.64 at baseline, 1.45 at 1 year, and 1.74 at 5 years. The negative predictive value for advanced fibrosis at a low cutoff point was 90.4/90.1 at baseline/1 year. Its specificity at a high cutoff point increased from 65.0% at baseline to 82.3% at 1 year. Multivariate analysis identified the FIB-4 index at 1 year as a predictive factor for a FIB-4 index > 2.67 at 5 years. A FIB-4 index < 1.30 was acceptable for excluding advanced fibrosis at baseline. In contrast, to evaluate and predict advanced liver fibrosis with the FIB-4 index at a high cutoff point, we should use the index at 1 year after appropriate therapy.

## 1. Introduction

Nonalcoholic fatty liver disease (NAFLD) is the most common chronic liver disease in Japan, and it affects up to 25–30% of the general adult population worldwide [1]. NAFLD is one of the phenotypes of metabolic syndrome in the liver, and it encompasses a wide spectrum of liver pathology, ranging from non-alcoholic fatty liver (NAFL), which is usually benign, to non-alcoholic steatohepatitis (NASH), which is characterized by steatosis, lobular inflammation, and hepatocellular injury, and may progress to liver cirrhosis (LC), hepatic failure, and hepatocellular carcinoma (HCC) in the absence of significant alcohol consumption. A recent large cohort study reported that hepatic fibrosis was the only predictive factor of not only liver-related events but also the mortality of patients with NAFLD [2,3]. These results indicate that accurate staging of fibrosis is clinically important for patients with NASH to prevent severe complications. Patients with NAFLD, particularly those with advanced symptoms, are at high risk for HCC and death from liver-related causes. Previous studies reported that 27.0–37.6% of patients with NAFLD experience disease progression [4,5,6,7,8,9,10,11]. Bodyweight gain, high insulin resistance, type 2 diabetes mellitus (T2DM), and low initial fibrosis stage are correlated with fibrosis progression among NAFLD patients [5,6,7]. Among NASH patients, obesity, change in alanine aminotransferase (ALT) levels, and weight gain are associated with progression [8,9]. One previous meta-analysis of serial biopsy studies reported that lobular inflammation at first biopsy predicted progression to advanced fibrosis [10]. However, no reports of noninvasive fibrosis score have clarified the predictive factors associated with histological changes in Japanese patients with biopsy-proven NAFLD.

Liver biopsy remains the gold standard for the diagnosis of NASH. However, liver biopsy is invasive, and has several limitations, such as sampling error and cost [12,13,14]. Intraobserver and interobserver variability are also potential concerns. In the previous study by Kleiner et al., eight pathologists reviewed the biopsy specimens twice. The intraobserver agreement rate for fibrosis was 0.85, and the interobserver agreement rate was 0.84 [15]. Ratziu et al. estimated the sampling error and intraobserver difference by collecting two specimens from each patient and having one pathologist review the specimens on two separate occasions, 3 months apart. There was at least a one-stage difference between the two specimens in 41% of patients. The reliability rate was only 0.47 [16]. Fibrosis variability was present not only in the early stage but also in the advanced stage. Non-invasive approaches to identify the stage of liver fibrosis have been devised with a combination of clinical parameters and imaging methods, including transient elastography (TE) or magnetic resonance elastography (MRE). However, obesity can be a reason for failed TE, and MRE is still expensive and not widely available. The diagnostic accuracy of early stage NASH (stage 1) from stages 2–4 is still unclear for both TE and MRE methods. The Fibrosis-4 (FIB-4) index is a noninvasive fibrosis score based on blood parameters measured routinely, such as aspartate aminotransferase (AST), ALT, and platelet count, as well as age. According to some studies, the performance of the FIB-4 index for the diagnosis of advanced fibrosis in NAFLD is better than that of other non-invasive parameters in both Caucasian and Japanese subjects [17,18]. However, the FIB-4 index often changes in the follow-up period, and its accuracy for detecting advanced fibrosis is not high enough. Furthermore, recent large cohort study in the general population reported that biomarkers including the FIB-4 index have limitations on their ability to evaluate liver fibrosis because of underlying disease. They found only modest associations between liver stiffness measured by TE and biomarkers of liver fibrosis [19]. There is a paucity of longitudinal follow-up data regarding changes in the FIB-4 index in patients with NAFLD. 

In this study, we therefore aimed to clarify the accuracy of the FIB-4 index at baseline and after appropriate therapy in the follow-up period, and to determine a strategy for detecting advanced fibrosis in patients with NAFLD using the FIB-4 index.

## 2. Materials and Methods

### 2.1. Patients

A total of 272 consecutive patients who underwent liver biopsy and were diagnosed with NAFLD at the Department of Gastroenterology and Hepatology, Kyoto Prefectural University of Medicine (Kyoto, Japan), and Center for Digestive and Liver Disease in Nara City Hospital (Nara, Japan) from January 2005 to March 2015 were enrolled in this retrospective study. All patients were followed up with for more than 5 years. We diagnosed NAFLD on the basis of liver biopsy findings of steatosis in at least 5% of hepatocytes and the exclusion of other liver diseases, including viral hepatitis, autoimmune hepatitis, and drug-induced liver disease. Patients with a daily alcohol consumption greater than 30 g for men and greater than 20 g for women were excluded. Of these, 52 patients underwent serial biopsies. The median interval between basal and second liver biopsies was 968 days (210–2778 days). Patients were given medications as necessary during the follow-up period. This study was approved by the Ethical Review Board of Kyoto Prefectural University of Medicine (ERB-C-544-2), approved on 10, July, 2012. Review Board of Nara City Hospital (No. 35), approved on 10 January 2016. All patients provided written informed consent at the time of liver biopsy, and the study was conducted in accordance with the 2013 Declaration of Helsinki.

### 2.2. Laboratory and Clinical Parameters

Venous blood samples were collected the morning after a 12-h overnight fast. Laboratory assays included blood cell counts and measurements of serum concentrations of AST, ALT, gamma glutamyl transpeptidase (GGT), total cholesterol, high-density lipoprotein (HDL) cholesterol, low-density lipoprotein (LDL) cholesterol, triglycerides, fasting plasma glucose (FPG), and type IV collagen 7s. These parameters were measured with standard clinical chemistry laboratory techniques. Body mass index (BMI) was calculated as weight in kilograms/(height in meters)^2^. T2DM was diagnosed according to the Report of the Expert Committee on the Diagnosis and Classification of Diabetes Mellitus or based on the administration of anti-T2DM agents. Patients with serum cholesterol concentrations > 220 mg/dl or triglyceride concentrations > 160 mg/dl or who were receiving treatment with anti-dyslipidemia agents were defined as having dyslipidemia. The Fibrosis-4 (FIB-4) index was calculated as follows: ([age (years) × AST (IU/ L)]/platelet count [10^9^/L]) × (ALT [IU/L])^1/2^.

### 2.3. Liver Histology

All enrolled patients underwent a percutaneous liver biopsy under ultrasonic guidance. The liver specimens were embedded in paraffin and stained with hematoxylin and eosin and Masson-trichrome. The specimens were evaluated by two hepatic pathologists who were blinded to the clinical findings. An adequate liver biopsy sample was defined as a specimen >1.5 cm long and/or having more than 11 portal tracts. NASH was defined as steatosis with lobular inflammation and ballooning degeneration, with or without Mallory–Denk bodies or fibrosis. Patients with liver biopsy specimens that showed simple steatosis or steatosis with non-specific inflammation were diagnosed with NAFL. Specimens with steatosis of <5, 5–33, >33–66, or >66% were scored as steatosis grades 0, 1, 2, and 3, respectively. For mild, moderate, and severe ballooning and inflammation (acinar and portal) the necroinflammatory grades were 1, 2, and 3, respectively. The severity of hepatic fibrosis (stage) was scored as: stage 1, zone 3 perisinusoidal fibrosis; stage 2, zone 3 perisinusoidal fibrosis with portal fibrosis; stage 3, zone 3 perisinusoidal fibrosis and portal fibrosis with bridging fibrosis; and stage 4, cirrhosis.

### 2.4. DNA Preparation and SNP Genotyping

Genomic DNA was extracted from blood samples via the DNeasy Blood & Tissue kit (Qiagen, Tokyo, Japan). The SNP rs738409 was genotyped in each sample using TaqMan SNP genotyping assays (Applied Biosystems, Foster City, CA, USA) with commercially available predesigned SNP-specific primers for PCR amplification and extension reactions according to the manufacturer’s protocol. The precise protocol was performed in a manner similar to that in our previous study [20,21].

### 2.5. Statistical Analysis

The distribution of subject characteristics was assessed by the chi-square test or Mann–Whitney’s U test, as appropriate. To assess the accuracy of the FIB-4 index in differentiating advanced fibrosis, we calculated the sensitivity (Se), specificity (Sp), positive predictive value (PPV), and negative predictive value (NPV) for the cut-off values (<1.30 and >2.67) proposed by Shah [18]. The dependent data was assessed by using the Wilcoxon signed-rank test. We compared fibrosis stage between first and second biopsy, and the change in fibrosis stage was divided by the interval period between basal and second biopsy in 52 patients. We performed logistic regression analysis to evaluate predictive factors of the FIB-4 index at 5 years adjusted for sex (male, female), age, BMI, presence of T2DM, hypertension, hyperlipidemia (yes, no), *PNPLA3* (CC, CG, GG), and FIB-4 index at baseline and 1 year. All reported P values were two-sided, and the significance level was set at 0.05. Statistical comparisons were performed with SPSS software (version 25, SPSS Inc., Chicago, IL, USA).

## 3. Results

### 3.1. Patient Characteristics

A total of 272 patients with NAFLD were analyzed. Table 1 summarizes the demographic profiles and laboratory and histologic data of the study patients at baseline and 5 years later. The median age was 60 years, 122 patients (44.9%) were men, 137 patients (50.4%) were diagnosed with T2DM, 184 (67.6%) had hypertension, and 184 (67.6%) had hyperlipidemia. Forty-six patients had advanced fibrosis (stage 3 or 4) including 16 patients with cirrhosis. There was a significant decrease in BMI and albumin, AST, ALT, GGT, and LDL-cholesterol levels from baseline to 5 years after treatment in the overall population. The mean FIB-4 indices for stages 0, 1, 2, 3, and 4 were 1.20, 1.59, 2.08, 2.44, and 3.31, respectively (*p* < 0.0001 by analysis of variance). The mean FIB-4 index was 1.55 in patients with stage 0–2 fibrosis and 2.72 in patients with stage 3–4 fibrosis (*p* < 0.001). Table 2 summarizes the demographic profiles and laboratory and histological data of study patients at initial and second biopsy. During the follow-up period, significant reductions in albumin, AST, ALT, γ-GT, platelet count, triglyceride, and type IV collagen 7s were observed. At initial biopsy, 14 patients had advanced fibrosis. At the second biopsy, 11 patients had advanced fibrosis, including 3 patients which had progressed to stage 4.

### 3.2. Correlation between Histological Change and Change in FIB-4 Index

**Figure 1:**
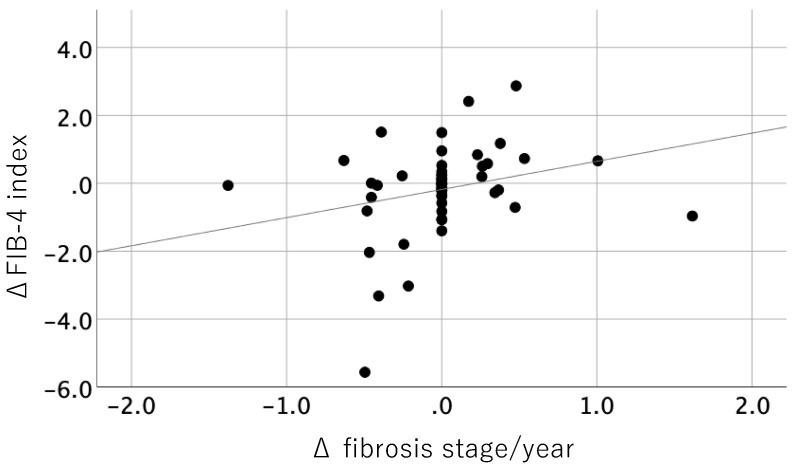
Correlation between changes in the FIB-4 index and change in fibrosis stage per year.

Among 52 patients, 13 patients (25%) had fibrosis progression of one stage or more, the stage remained unchanged in 26 patients (50%), and 13 patients (25%) showed fibrosis regression (Table 3). The change amounts of each fibrosis stage were ranged from −2 to 2 stages, and from −1.38 stage/year to 1.62 stage/year. The overall annual rate of fibrosis change was 0.002/year. Figure 1 shows the correlation between change in liver fibrosis stage per year and the FIB-4 index. There was significantly weak positive correlation between change in fibrosis stage and the FIB-4 index (*p* = 0.048, r = 0.265). Among 23 patients with a worse FIB-4 index result from baseline to the second biopsy, the fibrosis stage progression rate was 0.10 stage/year, while that of 29 patients with a better FIB-4 index result was −0.08 stage/year. Other fibrosis markers, such as platelet count and Type IV collagen 7s did not show a significant correlation. 

### 3.3. Changes in the FIB-4 Index During the Follow-Up Period and Accuracy for Advanced Fibrosis

**Figure 2:**
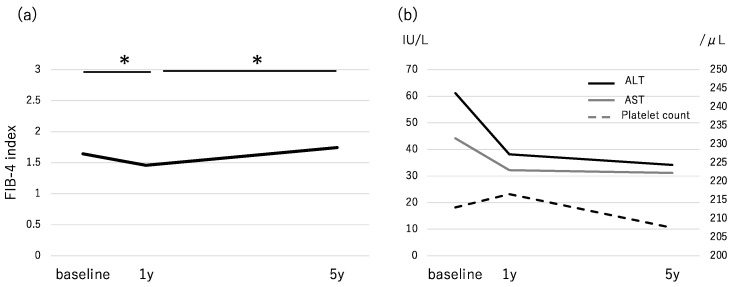
Changes in (**a**) the FIB-4 index and (**b**) AST, ALT, and platelet count in the follow-up period. * *p* < 0.05.

We divided subjects into three groups by cut-off values (<1.30, 1.30–2.67, and >2.67). The proportion of patients within each group (high/intermediate/low) was 39.7%/29.8%/30.5% at baseline and 23.5%/44.1%/32.4% at 5 years. The number of patients with a high FIB-4 index decreased from 108 at baseline to 64 at 5 years. The median FIB-4 index decreased significantly from 1.64 at baseline to 1.45 at 1 year (*p* < 0.001) and increased to 1.74 at 5 years (Figure 2a). Regarding the components of the FIB-4 index, the serum levels of AST and ALT decreased significantly from baseline to 1 year (*p* < 0.001). The level of ALT also decreased significantly from 1 year to 5 years (*p* = 0.017), while AST did not change significantly (*p* = 0.189). Platelet counts increased from baseline to 1 year, then decreased significantly from 1 year to 5 years (*p* = 0.007) (Figure 2b). Table 4 shows the number of patients according to the fibrosis stage at baseline and 1 year. Among the 46 patients with advanced fibrosis, 8 and 11 patients had a FIB-4 index result of <1.30 at baseline and at 1 year, respectively. Among 29 patients at stage 3,4 who had FIB-4 > 2.67 at baseline, 20 patients (69.0%) showed a FIB-4 > 2.67 at 1 year too. On the other hand, among 79 patients at stages 1,2 and with FIB-4 > 2.67 at baseline, only 40 patients (50.6%) showed FIB-4 > 2.67 at 1 year (Table 4). We calculated the sensitivity and specificity of the FIB-4 index at baseline and after 1 year for differentiating baseline advanced fibrosis from non-advanced fibrosis. At a low cut-off value, the sensitivity, specificity, positive predictive value (PPV), and negative predictive value (NPV) were 82.6%/33.2%/20.1%/90.4%, respectively, at baseline, and 76.9%/44.2%/21.7%/90.1%, respectively, at 1 year. At a high cut-off value, those values were 63.0%/65.0%/26.9%/89.6%, respectively, at baseline and 43.4%/82.3%/33.3%/87.7%, respectively, at 1 year (Table 4). 

### 3.4. Factors Associated with a FIB-4 Index >2.67 at 5 Years

Among 272 patients with NAFLD, the number of patients with a high FIB-4 index decreased from 108 (39.7%) at baseline to 64 (23.5%) at 5 years. We performed multivariate analysis using sex, age, hypertension, T2DM, hyperlipidemia, PNPLA3 genotype, the FIB-4 index at baseline, and FIB-4 index at 1 year as factors (Table 5). The analysis identified FIB-4 index at 1 year (>2.67, odds ratio [OR] 41.65, *p* < 0.01) as a predictive factor for a FIB-4 index > 2.67 at 5 years. On the other hand, the FIB-4 index at baseline did not predict a high FIB-4 index at 5 years. Among the 108 patients with a high FIB-4 index at baseline, 51 (47.2%) had a FIB-4 index > 2.67 at 5 years. Among 60 patients with a high FIB-4 index at 1 year, 49 (81.7%) had a FIB-4 index > 2.67 at 5 years.

## 4. Discussion

Staging of liver fibrosis in patients with NAFLD is essential for stratifying patients according to prognosis, treatment strategy, and guiding surveillance for the development of HCC. Because of the large number of patients with NAFLD, it is impossible to perform liver biopsy in all patients. Non-invasive fibrosis assessments are widely developed for the selection of patients considered to require further inspection. It is still controversial whether a non-invasive fibrosis score is preferred to elastography or not. Elastography has high diagnostic performance in noninvasive detection of liver fibrosis and steatosis in patients with NAFLD, especially with measuring spleen stiffness [22,23]. However, Liver stiffness measurement by TE is reported to be overestimated by transaminases flare [24]. Several studies suggested that FIB-4 index is considered to be one of the most useful scoring systems, especially combined with ultrasound and MRI-based elastography [25,26]. Sumida et al. reported that the FIB-4 index had a high NPV for excluding advanced fibrosis and was superior to other scoring systems [17]. The FIB-4 index also has several concerns. Whether the changes in the FIB-4 index during the follow-up period truly reflect a change in fibrosis is unclear. EASL-ALEH clinical practice guidelines reported that a FIB-4 index assessment may be less sensitive in detecting changes in intermediate stages of fibrosis [27]. One of the reasons for that concern is the components of the FIB-4 index. The serum levels of AST and ALT change drastically with diet and exercise therapy. Several treatments for complications of NAFLD, such as T2DM and hyperlipidemia, also decrease transaminase levels. Sodium glucose cotransporter 2 inhibitors and glucagon-like peptidase-1 receptor agonist use for T2DM can decrease transaminase activity in patients with NAFLD [27,28,29,30]. Statin use for hyperlipidemia was reported to have a protective effect against liver damage in patients with NAFLD [31]. It is also unknown whether the FIB-4 index can become a surrogate marker for evaluating histological changes, because longitudinal studies about the correlation between change in FIB-4 index and fibrosis stage are rarely performed.

In the present study, we first clarified the significance of determining the FIB-4 index to estimate fibrosis progression by serial biopsies study. The change in FIB-4 index was significantly correlated with change in fibrosis stage in Japanese patients with NAFLD. Our result indicate that serial FIB-4 index measurements are relevant for monitoring histological changes and have potential for good index in the treatment of NAFLD. The post hoc analysis of FLINT study revealed that a reduction in the FIB-4 index at 24 weeks significantly correlated with improvement of fibrosis stage at 72 weeks [32]. This report supported our results. 

The median level of AST/ALT decreased from 44/62 at baseline to 32/38 at 1 year and 31/34 at 5 years. It is hard to believe that fibrosis was ameliorated after 1 year of treatment. We previously reported that the average annual rate of progression of liver fibrosis was 0.002 stages per year in treated patients with NASH [33]. A meta-analysis of serial biopsy studies in mostly untreated patients with NAFLD identified the rate of fibrosis change to be 0.07 stages/year in NAFL and 0.14 stages/year in NASH [34]. Based on these studies, the fibrosis stage at 1 year after baseline may not change from that of baseline. The accuracy of the FIB-4 index in this study was worse than that in a previous study in Japanese patients [17]. A plausible reason for this difference may be the difference in cut-off value and the proportion of patients with advanced-stage disease. In this study, the accuracy of the FIB-4 index differed between baseline and 1 year. The NPV for a low cut-off value (<1.30) was about 90% and did not change between the two points. Regarding the high cut-off value (>2.67), among 108 patients who had a FIB-4 index > 2.67 at baseline, 79 (73.1%) had non-advanced fibrosis NAFLD. At 1 year, 40 of 60 patients (66.7%) who had a FIB-4 index > 2.67 were without advanced fibrosis. The specificity for diagnosing advanced fibrosis increased from 65% at baseline to 82.3% at 1 year. This result suggests that when we try to find patients with advanced fibrosis by using a high cut-off of the FIB-4 index, we should diagnose them after adequate therapy for NAFLD. We also could avoid unnecessary liver biopsy by using high cut-off of FIB-4 index at 1 year. The FIB-4 index at 1 year after baseline was also identified as a predictive factor for a FIB-4 index > 2.67 at 5 years after baseline. This finding suggests that early changes in the FIB-4 index may help us in predicting patients with therapy resistance and progression of fibrosis. These patients may need serial liver biopsy to confirm the change in histological findings and to determine changes in treatment or surveillance plan.

This study has several limitations. It was a retrospective study. The number of subjects, especially with advanced fibrosis, was smaller than in previous Asian studies [17,35]. Further large-scale studies using serial liver biopsy will be required to draw firm conclusions about the relationship between the FIB-4 index and liver fibrosis. Referral bias and patient selection bias could have existed because liver biopsy might have been considered for patients with NAFLD who were likely to have liver fibrosis. 

In conclusion, the NPV of a FIB-4 index < 1.30 was not different between baseline and 1 year; thus, it was acceptable to exclude advanced fibrosis at baseline. On the other hand, to evaluate and predict advanced liver fibrosis using a FIB-4 index > 2.67, we should use the index at 1 year after appropriate therapy. Taking the right opportunity for evaluating liver fibrosis by using the FIB-4 index seems to be important for excluding or predicting advanced fibrosis and avoiding unnecessary liver biopsies. 

## Figures and Tables

**Table 1:** Characteristics of 272 patients with nonalcoholic fatty liver disease at baseline and 5 years later.

Variable *n* = 272	Baseline	1 Year	5 Years	*p* (Baseline and 5 Years)
Sex, male	122 (44.9%)			
Age, years	60 (21–83)			
*PNPLA3*, CC/CG/GG	35/93/87			
Hyperlipidemia	184 (67.6%)			
T2DM	137 (50.4%)			
Hypertension	112 (41.2%)			
BMI, kg/m^2^	26.9 (18.9–43.0)	26.5 (17.2–43.0)	26.4 (17.4–46.2)	<0.001
Albumin, g/dL	4.4 (3.1–5.3)	4.4 (3.0–5.6)	4.3 (2.5–5.6)	0.013
AST, IU/L	44 (12–217)	32 (14–166)	31 (11–158)	<0.001
ALT, IU/L	62 (7–266)	38 (4–278)	34 (3–180)	<0.001
GGT, IU/L	61 (14–533)	47 (15–415)	39 (8–405)	<0.001
Platelet count, ×10^3^/μL	213 (46–454)	217 (46–393)	207 (25–433)	0.089
Total cholesterol, mg/dL	202 (66–350)	202 (74–334)	198 (87–301)	0.061
Triglycerides, mg/dL	146 (18–739)	140 (32–692)	132 (37–593)	0.179
LDL-C, mg/dL	124 (44–301)	120 (48–286)	118 (38–232)	0.007
HDL-C, mg/dL	54 (21-151)	54 (23–138)	54 (23–134)	0.490
FPG, mg/dL	102 (60–420)	106 (68–258)	111 (62–279)	<0.001
FIB-4 index	1.64 (0.31–9.84)	1.45 (0.32–11.73)	1.74 (0.31–15.86)	0.099
Type IV collagen 7s, ng/mL	4.6 (2.6–11.0)	4.8 (2.8–17.0)	4.8 (1.6–17.0)	0.557
Fibrosis stage (0/1/2/3/4)	83/99/44/30/16			

Results are presented as n (%) for qualitative data or as median (range) for quantitative data. Abbreviations: T2DM, type 2 diabetes mellitus; BMI, body mass index; AST, aspartate aminotransferase; ALT, alanine aminotransferase; GGT, gamma-glutamyl transferase; LDL-C, low-density lipoprotein cholesterol; HDL-C, high-density lipoprotein cholesterol; FIB-4, Fibrosis-4; FPG; fasting plasma glucose.

**Table 2:** Characteristics of 52 patients with nonalcoholic fatty liver disease at the initial and second biopsies.

Variable *n* = 52	At Initial Biopsy	At Second Biopsy	*p*
Sex, male	22 (42.3%)		
Age, years	68 (21–83)		
Hyperlipidemia	33 (63.5%)		
T2DM	26 (50.0%)		
Hypertension	24 (46.2%)		
BMI, kg/m^2^	26.4 (18.9––43.0)	26.6 (19.0–49.5)	0.486
Albumin, g/dL	4.3 (3.2–4.9)	4.1 (3.4–5.1)	0.001
AST, IU/L	57 (22–186)	38 (15–211)	<0.001
ALT, IU/L	83 (20–358)	46 (7–251)	<0.001
GGT, IU/L	73 (22–306)	45 (8–316)	0.009
Platelet count, ×10^3^/μL	207 (105–417)	195 (93–403)	0.040
Total cholesterol, mg/dL	201 (117–292)	191 (93–436)	0.203
Triglycerides, mg/dL	153 (71–559)	130 (60–351)	0.022
LDL-C, mg/dL	104 (43–225)	124 (43–209)	0.035
HDL-C, mg/dL	50 (22–85)	51 (28–95)	0.748
FPG, mg/dL	94 (60–260)	96 (65–182)	0.664
FIB-4 index	2.12 (0.31–9.04)	2.20 (0.26–3.14)	0.973
Type IV collagen 7s, ng/mL	5.3 (2.7–13.0)	4.4 (2.6–9.5)	0.002
Fibrosis stage (0/1/2/3/4)	0/24/14/14/0	2/18/21/8/3	

Abbreviations are defined in Table 1.

**Table 3:** Distribution of fibrosis stage at initial and second biopsies.

	Stage at Second Biopsy					
	0	1	2	3	4	Total
Stage at initial biopsy						
1	2	13	7	2	0	24
2	0	4	8	1	1	14
3	0	1	6	5	2	14
Total	2	18	21	8	3	52

**Table 4:** (a) Number of patients according to baseline fibrosis stage and (b) accuracy of the FIB-4 index at baseline and after 1 year.

**(a)**					
	**Baseline**		**1 Year**		
	Stage 3,4	Stage 0–2	Stage 3,4	Stage 0–2	
<1.30	8	75	11	100	
≧1.30	38	151	35	126	
≦2.67	17	147	26	186	
>2.67	29	79	20	40	
**(b)**					
		**Sensitivity**	**Specificity**	**PPV**	**NPV**
Baseline	Low cut-off	82.6	33.2	20.1	90.4
	High cut-off	63.0	65.0	26.9	89.6
1 year	Low cut-off	76.9	44.2	21.7	90.1
	High cut-off	43.4	82.3	33.3	87.7

Abbreviations: PPV, positive predictive value; NPV, negative predictive value.

**Table 5:** Factors associated with FIB-4 > 2.67 at 5 year in patients with non-alcoholic fatty liver disease based on univariate and multivariate analysis.

Variable	Category	*p* Value	OR (95% CI) ^a^	*p* Value
Age, year	Per 1 year		1.05 (0.99–1.11)	0.086
BMI, kg/m^2^	Per 1 kg/m^2^		0.97 (0.86–1.11)	0.679
Sex	1: male	0.002	0.34 (0.11–1.05)	0.060
	2: female			
T2DM	1: no	0.089	0.86 (0.31–2.36)	0.765
	2: yes			
*PNPLA3*	1: CC		1	
	2: CG	0.088	1.89 (0.31–11.41)	0.490
	3: GG	0.040	4.50 (0.74–27.32)	0.102
FIB-4 index at baseline	1: −2.67	<0.01	2.61 (0.88–7.69)	0.082
	2: >2.67			
FIB-4 index at 1 year	1: −2.67	<0.01	41.65 (13.11–132.33)	<0.01
	2: >2.67			

Abbreviations are defined in Table 1. OR: odds ratio, CI: confidence interval. ^a^ Estimated using logistic regression analysis.
